# Progress in Gynæcology

**Published:** 1900-05-26

**Authors:** 


### Progress in Gynæcology.

(*Continued from page* 104.)

**The Operative Treatment of Uterine Fibroids.**—Herman,[5] in a paper on this subject, says that the chief reasons calling for operative interference are hæmorrhage, large size of the tumour, pressure symptoms, and malignant degeneration. As to which is the best operation, he points out that there are many different methods, and each has its own sphere. If possible, the tumour itself should be removed, and the uterus left. He recommends the vaginal route if the tumour is not larger than a child's head, and can be pressed down into the pelvis; the advantages over the abdominal operation being that there is less shock, no liability to ventral hernia, and that it is easier to separate the bladder and ureters from the uterus from below than it is from above. If the abdomen has to be open the best operation is supravaginal amputation of the uterine body; enucleation with preservation of the uterus being advisable only in a very few cases. He strongly recommends the intraperitoneal method of dealing with the stump. Comparing supravaginal amputation of the body with panhysterectomy, he is in favour of the former operation, and thinks the only case in which there is any advantage in removing the cervix and body is when there is reason, from the rapid growth and vascularity of the tumour, to think that it is a sarcoma. In removing the cervix as well as the body of the uterus the ureters are more liable to be damaged, as the uterine arteries have to be tied further from the cervix; then, again, the cervix forms the centre of the pelvic floor. Gow[6] also strongly recommends supravaginal amputation of the body with intraperitoneal treatment of the stump in preference to all the other abdominal operations for fibroids. As regards oöphorectomy, Herman thinks it is inferior to removal of the fibroids or the part of the uterus from which the fibroid has grown. Formerly, when the mortality from oöphorectomy was much less than that of hysterectomy, the operation was much oftener indicated than at present; since, however, the mortality of hysterectomy has been so much reduced, oöphorectomy is not often called for, more especially as we know it is sometimes followed by grave mental troubles. As regards electrical treatment, his experience leads him to doubt whether electricity has any beneficial effect. Schauta[7] draws the following conclusions with regard to these neoplasms: (1) Operative treatment is not legitimate except when they are a cause of trouble that cannot be conquered by other means; (2) vaginal total extirpation should be considered as the safest, and, in the long run, the most successful operation. It should be performed in all cases where the tumour does not extend above the level of the umbilicus, and when it can be easily drawn into the pelvis; (3) for large not easily-moveable tumours, wholly or partially intraligamentary, abdominal total extirpation should have the preference; (4) supravaginal amputation, with intraperitoneal treatment of the stump should, he thinks, be gradually set aside in favour of abdominal total extirpation; (5) in emergency cases supravaginal amputation with extraperitoneal treatment of the stump, affords facility for speedy and absolute extraperitoneal execution, and offers an advantage not to be underrated in cases of extreme anæmia, asphyxia, weakness of the heart, and suppuration or necrosis of the tumour; (6) vaginal enucleation of broad-based, submucous tumours, either by way of the dilated cervix, or by the vaginal fornix after anterior or posterior kolpotomy, should be resorted to only in cases where there are special indications. Myomata being generally multiple, it would not be likely that the operation would afford durable results; (7) castration should be strongly objected to on the ground of its not bearing comparison with the radical operations with regard to reliability and immunity from danger. Doyen[8] considers that total abdominal hysterectomy is the operation of choice for all large fibroid tumours. Delagenieri advocates ligature of the uterine arteries in certain cases of fibroids. By this means hæmorrhage, he says, is often checked, and frequently the tumours diminish in size. Sinclair[9] recommends supravaginal amputation, with intraperitoneal treatment of the stump; but considers that the vaginal route should be selected whenever possible, inasmuch as the abdominal operation is a more grave proceeding. Recovery is more rapid if one or both ovaries are allowed to remain.

**Prolapsus Uteri.**—Herman[10] points out that, although in speaking of prolapsus, we call it prolapsus uteri, it is, as a matter of fact, prolapsus of a great deal more than the uterus, although that viscus is the most conspicuous part which comes down; usually there is prolapse not only of the uterus, but of the bladder, and Douglas's pouch, and of the vagina. The reason of this displacement is weakness of the structures which support the uterus, the most important of which are the pelvic fascia and the levator ani muscle. The great symptoms of prolapse are backache and bearing-down pain, and the characteristic feature of the pain is that it goes away when the patient lies down and pushes back the uterus. It is, he says, common to find women who, when they get lowered in nervous tone from any cause, get backache and bearing-down pain, which, no doubt, is due to temporary weakness of the uterine supports; when their general health is improved these symptoms disappear without any local treatment. In other cases, however, the amount of prolapsus is too great to be cured by general tonic treatment, and other methods have to be adopted. For these cases Herman recommends first of all the use of pessaries, Hodge, ring, Zwancke, or if these cannot be retained, one of the various forms of cup and stem pessary. If these methods are unsatisfactory, the question arises whether anything more can be done, and Herman discusses four operations that have been done for the cure of prolapse, viz., hysterectomy, Alexander's operation, colporrhaphy, and ventral fixation. The two first he considers are useless, because after hysterectomy the patient will still have prolapse of the bladder; and after Alexander's operation the uterus, instead of coming down lying back, comes down lying forward. Herman thinks the best results are obtained by a combination of colporrhaphy and ventral fixation. In performing ventral fixation, he lays stress on the importance of suturing the uterus to the abdominal muscles, rather than to the peritoneum. In cases where the uterus has been prolapsed long and exposed to friction, the cervix becomes swollen, œdematous, and ulcerated; in these cases it is best to begin by amputating the vaginal portion. Monao Lesser[11] recommends ventral fixation of the round ligament for retroversion and prolapsus uteri. It leaves the uterus in its free position, and neither interferes with its physiological requirements, nor changes to any material extent its relation with other organs. T. A. Helme[12] reports two cases of extirpation of the uterus for extreme hypertrophy and prolapse. The patient had suffered for many years from prolapse of the uterus along with the anterior vaginal wall and bladder. The uterus was removed in the usual way by ligaturing the broad ligaments in section. The peritoneal cavity was completely closed by careful suturing. The cut edges of the vaginal walls were then sutured, a broad surface being united. At a second operation the perineum was repaired, and the vaginal outlet narrowed. The results were quite satisfactory; the patient walked well, had no bladder trouble, and did not require a pessary.

**Vaginal Hysterectomy for Carcinoma Uteri.**—Bowerman Jessett,[13] in deciding whether vaginal hysterectomy should be performed in any given case of carcinoma uteri, is guided by the following rules: (1) If on examination of a patient at or past the menopause who is suffering from a discharge, which discharge is coloured and offensive, and in whose case the introduction of a sound causes bleeding, he would feel pretty certain that he had to deal with a case of carcinoma of the body of the uterus, and would advise operation. Should any doubt, however, exist, he would dilate the cervical canal, curette the cavity, and have the *débris* examined microscopically. If the report of the pathologist was unfavourable, he would at once urge operative measures for the removal of the whole organ. (2) If the disease had commenced in the cervical canal, or the external os, even if the walls of the vagina were somewhat invaded, so long as the uterus itself was not fixed or the broad ligament invaded, he would not hesitate to advise operation. Even in advanced cases, so long as the uterus is movable, he is convinced that much relief may be afforded and life may be prolonged by vaginal hysterectomy. From post-mortem examinations of a large number of cases of cancer of the uterus, he is convinced that the iliac glands are not affected until much later in the disease than is generally supposed.

[5] Practitioner, Dec., 1899. [6] Lancet, Jan, 13, 1900. [7] Inter. Med. Mag., Oct., 1899. [8] Med. Chron, Oct., 1899. [9] Med. Chron., Oct., 1899. [10] Clin. Jour., Dec. 13, 1899. [11] Med. Rec., Oct. 14, 1899. [12] Brit. Med. Jour., Dec. 9, 1899. [13] Lancet, Nov. 8, 1899.

